# Contrasting Impacts of Targeted Disruption of the Cancer Stem Cell Marker CD133 and Its Epigenetic Regulator TRIM28 in Colorectal Cancer Cells

**DOI:** 10.3390/ijms262210862

**Published:** 2025-11-09

**Authors:** Irina V. Kholodenko, Aleena A. Saidova, Daria M. Potashnikova, Viktoriia A. Arzumanian, Daniil D. Romashin, Anna V. Tvorogova, Ekaterina V. Poverennaya, Konstantin N. Yarygin, Yan S. Kim

**Affiliations:** 1 V.N. Orekhovich Institute of Biomedical Chemistry, 119121 Moscow, Russia; irkhol@yandex.ru (I.V.K.); arzumanian.victoria@gmail.com (V.A.A.);; 2 Biology Department, Shenzhen MSU-BIT University, Shenzhen 517182, China; 3 Department of Cell Biology and Histology, School of Biology, M.V. Lomonosov Moscow State University, 119234 Moscow, Russia; 4 Center of Genomic Sciences, Institute of Gene Biology, Russian Academy of Sciences, 119334 Moscow, Russia

**Keywords:** cancer stem cells, CD133, TRIM28, cancer heterogeneity

## Abstract

Cancer stem cells (CSCs) play a crucial role in colorectal cancer by sustaining intratumoral heterogeneity, therapeutic resistance, and metastatic potential. CD133 (PROM1) is among the most frequently used surface markers for CSC identification, whereas TRIM28, a versatile epigenetic regulator, has been implicated in controlling CD133 expression and stem-like features. In this study, we performed a detailed molecular and functional analysis of Caco2 colorectal cancer cell clones with individual knockouts of CD133 or TRIM28. Elimination of CD133 neither altered global gene expression, as confirmed by transcriptome profiling, nor affected key cellular properties. In contrast, loss of TRIM28 led to a marked reduction in CD133 protein abundance and induced extensive molecular and phenotypic remodeling. TRIM28 knockout was associated with broad transcriptomic changes involving more than 500 differentially expressed genes, decreased proliferative activity monitored by time-lapse imaging, and reduced sensitivity to paclitaxel, cisplatin, and curcumin. Furthermore, immune evasion molecules CD24 and CD47 (“don’t eat me” signals) were strongly upregulated in TRIM28-deficient cells, consistently confirmed by both RNA-Seq and flow cytometry analyses. At the same time, imaging flow cytometry and mitochondrial activity assays indicated that these effects were not due to major shifts in mitotic index or bioenergetic status. Altogether, our results demonstrate that TRIM28, rather than CD133, functions as a central regulator of CSC-associated phenotypes in colorectal cancer. These findings highlight the importance of epigenetic context in CSC biology and may inform the development of more effective therapeutic strategies.

## 1. Introduction

The cancer stem cell (CSC) hypothesis emerged in the late 1990s, primarily through the work of Bonnet and Dick, who proposed that the hierarchical cellular organization observed in normal ontogeny, with stem cells residing at the apex, might also be applicable to tumor architecture [1]. This notion suggested that tumors, like tissues, are sustained by a rare population of cells with stem-like properties. However, more recent evidence challenges the existence of such a strict hierarchy within tumors [2]. Instead, it appears that the observed cellular heterogeneity may arise from the phenotypic plasticity of cancer cells, allowing for the emergence of subpopulations with diverse intrinsic biological properties—including differences in proliferation, invasion, migratory potential, drug resistance, and gene expression profiles [3]. Importantly, these differences are often driven by post-genomic alterations, not genomic mutations, a phenomenon studied within the framework of clonal evolution theory [4].

Nonetheless, the foundational studies that shaped the CSC concept made a critical contribution by demonstrating the intratumoral heterogeneity and, crucially, the potential to study this heterogeneity through surface markers, some of which we have previously described in our review [5]. The expression of such markers has been shown to correlate with the key biological features of distinct tumor subpopulations [6]. This makes them not only valuable tools for dissecting tumor biology, but also promising targets for diagnostic and therapeutic strategies [7]. Therefore, the so-called CSC population may best be understood not as a distinct stem cell lineage but rather as a dynamic subpopulation of tumor cells with the most aggressive phenotype. This interpretation is supported by various in vitro and in vivo studies that demonstrate enhanced tumorigenic capacity, resistance to therapy, and metastatic potential in cells bearing the so-called CSC profile [3,6].

One of the most commonly used markers for CSCs is CD133, which has been widely employed to identify and isolate this population across various tumor types [5,8]. Its expression has been shown to correlate with a number of adverse clinicopathological features in different cancers [9]. Despite the extensive data on CD133 interaction partners [10] and the molecular pathways affecting its function [11,12], the specific cellular function of CD133 within the cell remains unclear. Moreover, the application of surface markers such as CD133 for targeted therapy is hindered by the non-specificity of their expression to tumor tissue and the inherent plasticity of the CD133+ phenotype itself [13,14]. Therefore, targeting key regulators of CD133-associated heterogeneity, which underlies the CSC phenotype, appears to be a more promising strategy.

Previously, we demonstrated that the transcriptional regulator TRIM28 (Tripartite Motif Containing 28), also known as KAP1 (KRAB-associated protein 1), is capable of modulating CD133 expression, as its knockout resulted in a significant reduction in CD133 levels in the Caco2 cell line [15]. In this study, we systematically compared the effects of individual knockouts of CD133 and TRIM28 in Caco2 cells on a range of fundamental cellular properties. These included assessments of cell proliferation, migration capacity, resistance to conventional chemotherapeutic agents, and sensitivity to apoptosis, alongside evaluation of mitochondrial membrane potential. Additionally, we performed RNA-Seq–based transcriptomic profiling and analyzed the expression patterns of a broad panel of membrane-associated markers representing distinct functional categories in the knockout cells compared to the wild-type Caco2 cells.

## 2. Results

### 2.1. TRIM28 Knockout, but Not CD133 Knockout, Reduces Proliferative Activity in Caco2 Cells, While Neither Affects Cell Migration

Since cancer stem cells (CSCs) exhibit high proliferative and migratory potential, it is important to assess the impact of knocking out CD133, the most commonly used classical surface marker of CSCs, and its expression regulator TRIM28 on these fundamental cellular functions. The generation of Caco2 clones with TRIM28 knockout (TRIM28-KO) and corresponding control clones has been described previously [15]. CD133 knockout (CD133-KO) clones were generated de novo following the procedure as described elsewhere [16]. The Caco2 cell clones used in this study were divided into three groups according to their genetic background. The Control group (reference clones) included clones S1, S2, S4, and S5. The CD133 knockout (CD133-KO) group consisted of clones C1S, C2S, C3S, and C4S. The TRIM28 knockout (TRIM28-KO) group comprised clones T10, T14, T47, T50, and T51.

**Figure 1:**
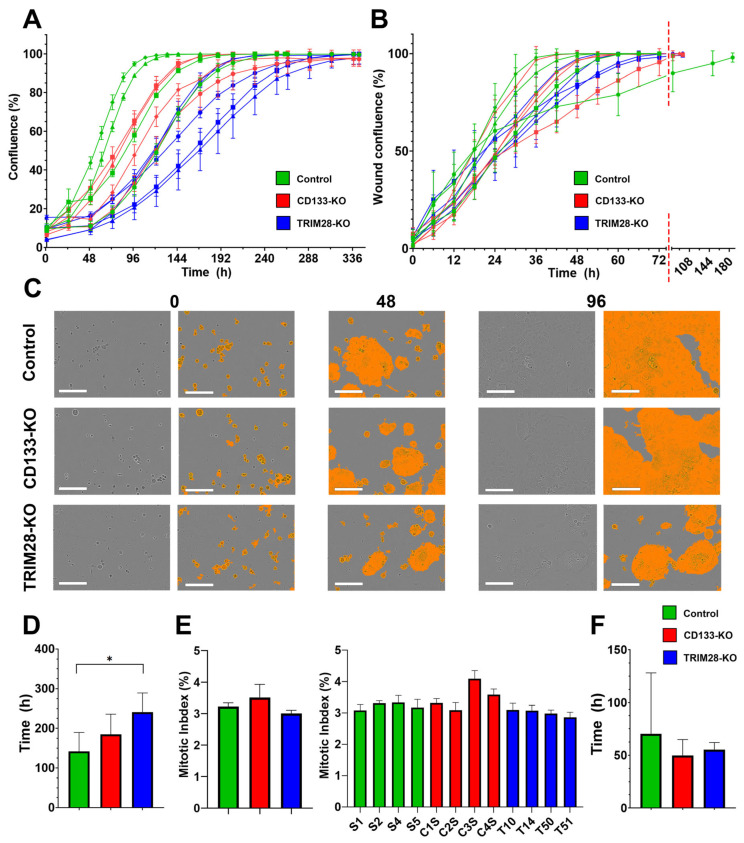
Cell proliferation and migration assessment in control Caco2 clones (Control group, green) and clones with CD133 knockout (CD133-KO group, red) or TRIM28 knockout (TRIM28-KO group, blue). (**A**) Growth curves and (**B**) wound confluence curves of different clone (each symbol represents the data for individual clone, mean ± SD). The red dashed line indicates a change in the time scale on the X-axis. (**C**) Representative brightfield images with confluence mask for time-lapse cell growth of three different Caco2 clones in three time points—0, 48 and 96 h. (**D**) Time required to reach confluence in proliferative activity assay (mean ± SD; Control: n = 4, CD133-KO: n = 4, TRIM28-KO: n = 5) and (**F**) time required for wound closure (mean ± SD; Control: n = 4, CD133-KO: n = 4, TRIM28-KO: n = 4). (**E**) Mean of mitotic index (MI) in three group of Caco2 clones (left panel) and mitotic indexes of individual clones (right panel). To improve the clarity of data presentation graph ((**E**), right panel) does not display statistical annotations for the significant differences in mitotic index between clone C3S and all other Caco2 clones, as well as between clone C4S and clones T50 and T51. * *p* ≤ 0.05; Scale bar—200 µm.

TRIM28-KO clones exhibited a statistically significant reduction in proliferation rate compared to the reference Control group clones (Figure 1A,C,D): ANOVA F(2,10) = 4.68, *p* = 0.037; Tukey’s post hoc test, *p* = 0.03 (Control vs. TRIM28-KO). The mean time required to reach confluence for the Control, CD133-KO, and TRIM28-KO groups was 141.4 ± 48.4, 184.9 ± 50.9, and 241.2 ± 48.1 h, respectively. Interestingly, despite the differences in proliferation rate mitotic index (MI) did not differ significantly among the three Caco2 clones groups (Figure 1E): ANOVA F(2,9) = 3.99, *p* = 0.06, with mean MI values of 3.22%, 3.51%, and 3.00% for the Control, CD133-KO, and TRIM28-KO groups, respectively. This apparent discrepancy between actual proliferation rates and the MI may be attributed, in part, to the limited number of analyzed clones, which may not provide sufficient statistical power to detect subtle differences in MI. Additionally, the MI does not always accurately reflect the true proliferative activity of cell populations. We also assessed MI levels across individual Caco2 clones separately. Statistical analysis revealed a significant difference in the MI level of clone C3S (ANOVA F(11,24) = 8.38, *p* < 0.0001) compared to all other clones (Tukey’s post hoc test *p* values range between 0.0001 and 0.004), and of clone C4S relative to clones T50 and T51 (Tukey’s post hoc test *p* < 0.05 and *p* < 0.01, respectively; Figure 1E). This result highlights the influence of clonal variability within groups.

Meanwhile no statistically significant differences were observed in average migration rates across the three Caco2 clone groups (Figure 1B,F): ANOVA F(2,9) = 0.38, *p* = 0.69. The mean wound closure times for the Control, CD133-KO, and TRIM28-KO groups were 70.5 ± 57.6, 49.9 ± 15.0, and 55.1 ± 7.0 h, respectively. The markedly slower migration observed in a single clone (S1) within the Control group again highlights high intragroup heterogeneity. To further characterize the functional consequences of TRIM28 and CD133 disruption, we next assessed the sensitivity of each group to various chemotherapeutic and cytotoxic agents.

### 2.2. TRIM28 Knockout Increases Resistance to Traditional Chemotherapeutic Agents in Caco2 Cells, While CD133 Knockout Has No Effect on Drug Sensitivity

Normal and particularly cancer stem cell populations are known for their intrinsic resistance to conventional chemotherapeutic agents, contributing to tissue homeostasis in the former, and to treatment failure and tumor relapse in the latter. Given that CD133 is a widely recognized surface marker of CSCs and TRIM28 is increasingly implicated in the regulation of stemness-associated pathways, we next examined whether knockout of either gene in Caco2 cells influences their sensitivity to various chemotherapeutic agents.

**Figure 2:**
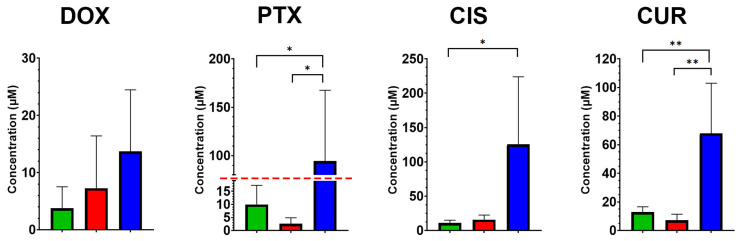
IC50 values for traditional chemotherapeutic agent of Control (green), CD133-KO (red) and TRIM28-KO (blue) Caco2 clones. The IC50 values (mean ± SD) are presented for all experimental groups, each comprising four independent clones for all treatments. The red dashed line indicates a change in the concentration scale on the axis. DOX—doxorubicin, PTX—paclitaxel, CIS—cisplatin and CUR—curcumin. * *p* ≤ 0.05, ** *p* ≤ 0.01.

MTT assay revealed significant differences in drug sensitivity among Caco2 clone groups, particularly in response to TRIM28 knockout. A statistically significant increase in IC50 values was observed for TRIM28-KO clones compared to both CD133-KO and Control clones for paclitaxel/PTX (Figure 2; ANOVA F(2,9) = 5.73, *p* = 0.025; Tukey’s post hoc test: *p* = 0.05 and *p* = 0.03, respectively) and curcumin/CUR (ANOVA F(2,9) = 10.8, *p* = 0.004; Tukey’s test: *p* = 0.006 and *p* = 0.01). In the case of cisplatin/CIS, TRIM28-KO clones also exhibited significantly higher IC50 values compared to the Control group (ANOVA F(2,9) = 5.18, *p* = 0.03; Tukey’s test: *p* = 0.05). No statistically significant differences were detected among the groups in response to doxorubicin/DOX (ANOVA F(2,9) = 1.42, *p* = 0.29). The mean IC50 values (in µM) for the Control/CD133-KO/TRIM28-KO groups were as follows. DOX: 3.78/7.28/13.7, PTX: 9.95/2.58/94.28, CIS: 10.98/15.75/125.55 and CUR: 12.93/7.12/68.00.

These results suggest that TRIM28 knockout is associated with a substantial more resistanсе to multiple cytotoxic agents, whereas CD133 knockout did not lead to consistent changes in drug response profiles. This reduced sensitivity to chemotherapeutic agents may stem from the lower proliferative activity observed in TRIM28-KO clones, as cells with diminished proliferation rates—such as quiescent cells—are generally less responsive to conventional cytotoxic drugs. To further investigate this possibility, we next examined whether the altered drug response could be linked to broader changes in mitochondrial function or apoptotic susceptibility, both of which are established modulators of chemotherapy efficacy.

### 2.3. Knockout of TRIM28 or CD133 Does Not Alter Mitochondrial Membrane Potential or Apoptotic Susceptibility of Caco2 Cells

Mitochondrial membrane potential was assessed by potential-dependent fluorescent dye MitoView 633. The results revealed no statistically significant differences in mitochondrial potential among the Control, CD133-KO, and TRIM28-KO groups, with mean relative fluorescence intensity (RFI) values of 2664.9, 724.7, and 807.7, respectively (ANOVA F(2,9) = 2.25, *p* = 0.16).

**Figure 3:**
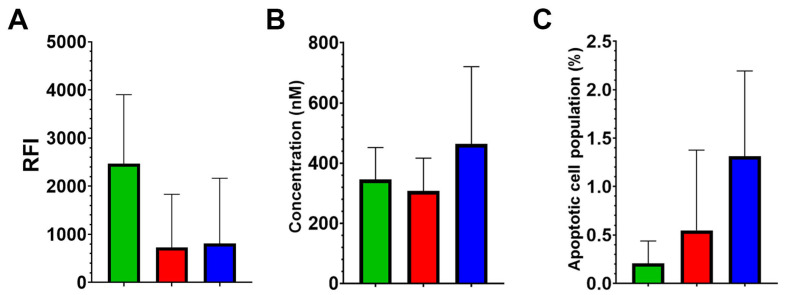
Mitochondrial membrane potential and apoptotic susceptibility of Control (green), CD133-KO (red) and TRIM28-KO (blue) Caco2 clones. (**A**) Mean of relative fluorescence intensity (±SD) of membrane potential-dependent MitoView 633 (mean ± SD; Control: n = 4, CD133-KO: n = 4, TRIM28-KO: n = 4). (**B**) IC50 concentration for staurosporine-induced cytotoxicity (mean ± SD; Control: n = 4, CD133-KO: n = 4, TRIM28-KO: n = 4). (**C**) Percentage of Annexin V-positive cells under standard culture conditions (mean ± SD; Control: n = 4, CD133-KO: n = 4, TRIM28-KO: n = 5).

To evaluate apoptotic susceptibility, cell viability in response to the classical apoptosis inducer staurosporine was measured using the MTT assay (Figure 3B). The IC50 values for staurosporine did not differ significantly among the three clone groups, with mean values of 345.6, 307.1, and 463.6 nM for the Control, CD133-KO, and TRIM28-KO groups, respectively (ANOVA F(2,9) = 0.89, *p* = 0.44).

Spontaneous apoptosis under standard culture conditions was also examined by quantifying AnnexinV-positive cell populations using FACS (Fluorescence-Activated Cell Sorting). No statistically significant differences were observed among the groups (ANOVA F(2,10) = 2.75, *p* = 0.11), indicating that baseline apoptotic levels were comparable across all clones (Figure 3C).

Together, these findings indicate that neither CD133 nor TRIM28 knockout substantially alters mitochondrial membrane potential or apoptotic susceptibility in Caco2 cells under standard conditions. Despite pronounced differences in proliferative behavior and chemotherapeutic sensitivity observed in TRIM28-KO clones, these effects do not appear to be mediated by major shifts in mitochondrial bioenergetics or baseline apoptosis levels.

To gain a more comprehensive understanding of the molecular mechanisms underlying the observed phenotypic changes, we next performed global transcriptomic profiling to uncover broader gene expression patterns associated with CD133 and TRIM28 loss.

### 2.4. TRIM28 Knockout Induces Broad Transcriptomic Alterations in Caco2 Cells, While CD133 Knockout Does Not Affect Gene Expression

**Figure 4:**
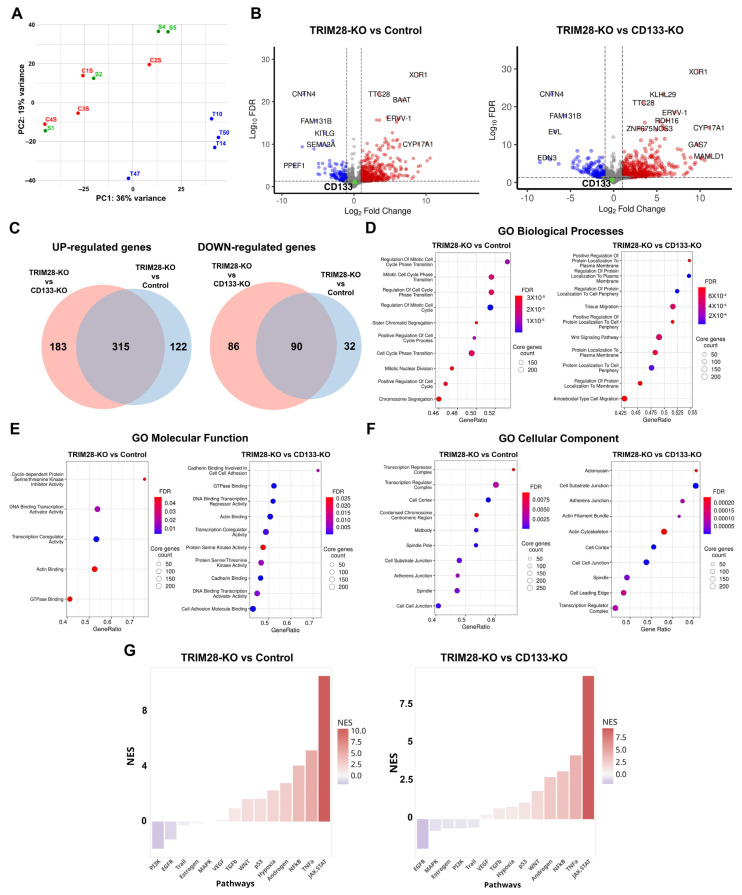
Comparative Whole-Transcriptome Profiling and GO Enrichment Analysis of Caco2 Control (n = 4), CD133-KO (n = 4), and TRIM28-KO (n = 4) clones. (**A**) Principal component analysis (PCA) plot showing clustering of Control (green), CD133-KO (red), and TRIM28-KO (blue) clones. (**B**) Volcano plots displaying differentially expressed genes (DEGs) in TRIM28-KO vs. Control and TRIM28-KO vs. CD133-KO comparisons. (**C**–**F**) Gene Ontology (GO) enrichment analysis of DEGs for the TRIM28-KO vs. Control and TRIM28-KO vs. CD133-KO comparisons: top terms from Biological Process, Molecular Function, and Cellular Component categories are presented. (**G**) PROGENy pathway analysis.

A comparative transcriptomic analysis was initially performed across twelve Caco2 clones by RNA-Seq. On average, 66,407,603 paired-end reads were generated per sample, with a mean read length of 202 bp. Quality metrics and the number of expressed genes for each clone are provided in Supplementary Table S1. Principal component analysis (PCA) revealed that Control and CD133-KO clones exhibited similar gene expression profiles, forming a single cluster, while TRIM28-KO clones segregated into a distinct cluster (Figure 4A). Given the absence of significant variation between Control and CD133-KO groups, differential gene expression (DEG) analysis was conducted only for TRIM28-KO vs. Control and TRIM28-KO vs. CD133-KO comparisons.

Differential expression analysis revealed the highest number of changes when comparing TRIM28-KO and CD133-KO samples, with a total of 674 differentially expressed genes (DEGs). Among these, 498 genes were upregulated and 176 downregulated in the TRIM28-KO group (Figure 4C). Comparison of TRIM28-KO clones with Controls identified 559 DEGs, including 437 upregulated and 122 downregulated transcripts. Interestingly, although the Control and CD133-KO clones clustered together in the PCA plot, the number of differentially expressed genes, both upregulated and downregulated, in these groups relative to TRIM28-KO was still large (Figure 4C). Among the genes upregulated in TRIM28-KO clones, 50.8% were shared between the comparisons with both Control and CD133-KO groups, whereas 43.3% of downregulated genes were common to both comparisons.

**Figure 5:**
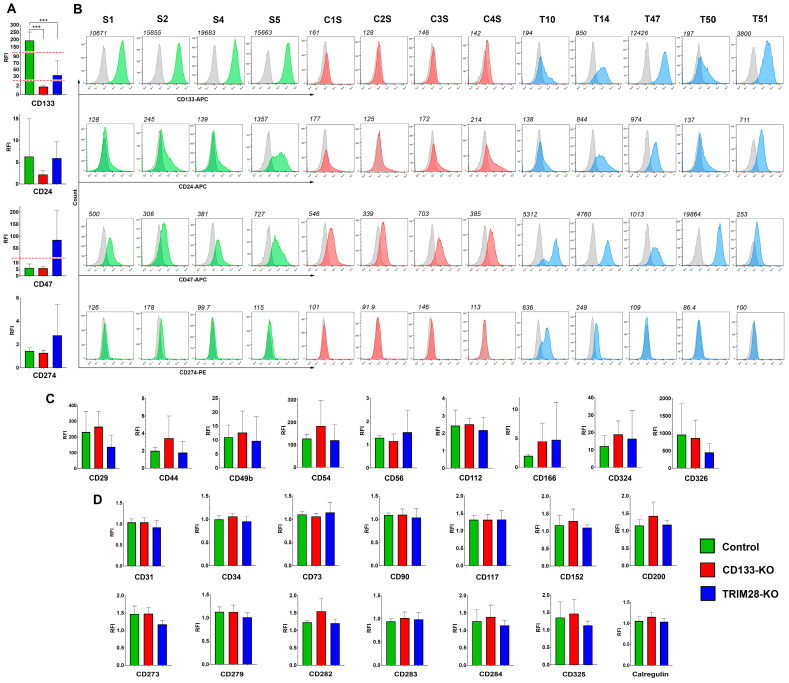
Flow cytometry analysis of surface marker expression in Control (green, n = 4), CD133-KO (red, n = 4), and TRIM28-KO (blue, n = 5) Caco2 clones. Grey color. (**A**) Quantification of selected markers by RFI whose expression showed notable correlation with CD133 or TRIM28 knockout status and (**B**) their representative FACS histograms. The red dashed line indicates a change in the RFI scale on the axis. Grey histograms represent autofluorescence of unstained cells; (**C**) Markers that exhibited detectable expression in at least one clone but showed no clear association with CD133 or TRIM28 knockout. (**D**) Markers with no detectable expression in any of the analyzed clones. *** *p* ≤ 0.001.

As expected, TRIM28 mRNA expression was significantly reduced in TRIM28-KO cells compared to both Control (~7.6-fold decrease; FDR < 0.001) and CD133-KO groups (~6-fold decrease; FDR < 0.001). In contrast, CD133 mRNA expression levels remained unchanged in all comparisons (Figure 4B), despite the absence of detectable CD133 protein in CD133-KO clones and its significant reduction in TRIM28-KO clones (Figure 5). These findings suggest that both the knockout and TRIM28-associated CD133 protein downregulation may be mediated through translational-level mechanisms rather than transcriptional changes.

Gene Ontology (GO) enrichment analysis of the TRIM28-KO vs. Control comparison revealed a marked overrepresentation of differentially expressed genes (DEGs) associated with cell cycle regulation. Notably, all of the top ten enriched GO Biological Process terms were directly related to cell division (Figure 4D), including “Regulation of Mitotic Cell Cycle Phase Transition” and “Mitotic Nuclear Division”.

Interestingly, among the differentially expressed genes (DEGs) enriched within these GO terms were genes with opposing roles in proliferation regulation. For example, PRAP1 (~11.1-fold increase; FDR = 0.004), which has been implicated in negative regulation of cell proliferation, and TGFA (~14.1-fold increase; FDR = 0.001), a well-characterized mitogen known to promote growth across various cell types, were both upregulated in TRIM28-KO clones. Given the markedly delayed monolayer formation observed in TRIM28-KO clones, as demonstrated by time-lapse imaging (Figure 1), it is plausible that the upregulation of mitogenic factors such as TGFA may reflect a compensatory response to the proliferation-inhibiting effects of TRIM28 depletion.

In addition, GO enrichment analysis of Molecular Function terms further substantiated the impact of TRIM28 knockout on cell cycle regulation, with significantly enriched terms such as “Cyclin-dependent Protein Serine/Threonine Kinase Inhibitor Activity” and “Actin Binding” (Figure 4E). Furthermore, GO Cellular Component enrichment analysis revealed an overrepresentation of DEGs localized to structures involved in cell division, including the “Condensed Chromosome Centromeric Region” and “Midbody”, further supporting a role for TRIM28 in regulating proliferative programs (Figure 4F). Among the differentially expressed genes (DEGs) identified within several of the enriched GO terms, CCR5 (~21.5-fold increase; FDR = 0.009), CXCR4 (~8.0-fold increase; FDR < 0.001), and ANXA8 (~2.6-fold increase; FDR = 0.03) stood out as regulators of cell proliferation. All of these genes were upregulated in TRIM28-KO clones, further suggesting their potential involvement in compensatory mechanisms regulating cell proliferation in the absence of TRIM28.

Interestingly, although Control and CD133-KO clones did not form distinct clusters in the PCA analysis, both groups exhibited sufficiently divergent DEGs profiles when compared to TRIM28-KO clones. These differences were further reflected in distinct patterns of Gene Ontology (GO) term enrichment, indicating that the transcriptomic responses to TRIM28 knockout differ markedly depending on the reference group used for comparison. Gene Ontology enrichment analysis for the TRIM28-KO vs. CD133-KO comparison revealed a prominent overrepresentation of GO Biological Process terms related to membrane localization. Among the most significantly enriched categories were terms such as “Regulation of Protein Localization to Plasma Membrane” and “Protein Localization to Cell Periphery”, indicating that many of the differentially expressed genes are involved in membrane-associated functions (Figure 4D). For instance, in TRIM28-KO clones, the genes ARHGAP44 (~24.3-fold increase; FDR < 0.001) and ZDHHC2 (~2.3-fold increase; FDR = 0.04) were upregulated. Both represent key candidates involved in the regulation of protein trafficking and anchoring at the plasma membrane, highlighting potential mechanisms through which TRIM28 knockout may influence membrane-associated signaling and cellular behavior.

Moreover, the DEGs set showed significant enrichment for GO Biological Process terms associated with cellular migration, including “Tissue Migration” and “Ameboidal-type Cell Migration”, suggesting that TRIM28 knockout may modulate genes involved in dynamic membrane remodeling and motility-related processes. In addition, GO enrichment analysis further revealed that the DEGs identified in the TRIM28-KO vs. CD133-KO comparison were significantly enriched for adhesion-associated categories. Specifically, GO Molecular Function terms included “Cadherin Binding Involved in Cell–Cell Adhesion” and “Cell Adhesion Molecule Binding” (Figure 4E). Correspondingly, GO Cellular Component terms highlighted structures related to adhesion and motility, such as «Cell–substrate junction» and “Adherens Junction”, with “Actomyosin” ranking among the top enriched terms, emphasizing the cytoskeletal components involved in migratory behavior (Figure 4F). Among the genes associated with migratory properties, particular attention should be given to the transcription factor SNAI2 (~19.7-fold increase; FDR = 0.01), as well as TGFB2 (~3.4-fold increase; FDR = 0.016) and TGFBR1 (~2.1-fold increase; FDR = 0.015), which are regulators of epithelial-to-mesenchymal transition (EMT), a process known to enhance cell migratory potential. In addition, other genes such as SPARC (~46.1-fold increase; FDR < 0.001), THBS1 (~2.9-fold increase; FDR = 0.013), and WNT7A (~5.0-fold increase; FDR = 0.04) were also upregulated, all of which are implicated in remodeling the extracellular matrix and promoting cellular motility. It is noteworthy that among the identified DEGs, THBS1 was also involved in adhesion, along with several other genes such as IGF1 (~60.7-fold increase; FDR = 0.0013) and classical cadherins—CDH11 (~32.5-fold increase; FDR = 0.003), CDH23 (~4.2-fold increase; FDR = 0.005), CDH6 (~5.4-fold increase; FDR = 0.037), as well as integrin subunits—ITGA6 (~2.1-fold increase; FDR = 0.026), ITGAV (~2.4-fold increase; FDR = 0.011), and ITGB6 (~2.2-fold increase; FDR = 0.039).

Since CD133 knockout did not result in detectable differential gene expression, it is plausible that the transcriptomic profile of CD133-KO clones closely reflects the baseline state of the parental Caco2 cell line. Therefore, all differentially expressed genes (DEGs) identified in both TRIM28-KO vs. Control and TRIM28-KO vs. CD133-KO comparisons can be considered as true DEGs associated with TRIM28 loss relative to the wild-type background. TRIM28 knockout in Caco2 cells induces pronounced transcriptomic alterations, prominently affecting genes involved in cell cycle regulation, adhesion, membrane localization, and migration. Importantly, the TRIM28-KO DEGs profile was enriched not only for general regulators but also for key stimulators of proliferation and motility, including mitogenic and EMT-associated factors most of which upregulated due TRIM28 loss. In contrast, CD133 knockout did not result in significant changes in gene expression, further highlighting the central role of TRIM28 in driving proliferative and migratory programs in Caco2 cells.

Pathway activity analysis using PROGENy revealed highly consistent alterations in TRIM28-deficient cells regardless of the chosen reference group—Control or CD133-KO (Figure 4G). In both comparisons, we observed a robust upregulation of inflammatory and stress-associated pathways, most prominently JAK/STAT, TNFα, and NFκB signaling, accompanied by moderate activation of p53, WNT, TGFβ, hypoxia, and androgen-related cascades. Conversely, proliferative signaling through EGFR, PI3K, and MAPK was consistently downregulated, suggesting a coordinated suppression of growth factor–driven programs. Notably, the magnitude of JAK/STAT and TNFα activation was higher in the TRIM28-KO versus Control comparison, whereas suppression of MAPK and estrogen pathways was more pronounced when CD133-KO clones served as the reference. These findings demonstrate that TRIM28 loss induces a reproducible shift from proliferative to pro-inflammatory signaling, in agreement with the transcriptomic reprogramming observed in RNA-Seq analysis and with functional evidence of reduced proliferation (Figure 1) and altered immune checkpoint expression in TRIM28-KO clones (see Section 2.5).

To further explore whether TRIM28 or CD133 knockout affects phenotypic identity and stemness, adhesion and immunity-associated surface properties of Caco2 clones, we next analyzed the expression of a wide range of cell surface markers by flow cytometry.

### 2.5. TRIM28, but Not CD133, Knockout Induces Modest Changes in Surface Marker Expression in a Clone-Dependent Manner in Caco2 Cells

Surface marker expression is of particular interest due to its potential applications in both cancer diagnostics and targeted therapy; therefore, we next analyzed whether the knockout of CD133 or its epigenetic regulator TRIM28 affects the membrane proteome of Caco2 cells. We analyzed a broad panel of surface molecules, including markers associated with cancer and normal stem cells, adhesion proteins, differentiation markers, as well as immune-related proteins such as toll-like receptors, immune checkpoint molecules, and “eat me”/“don’t eat me” signals (Figure 5).

The expression levels of 27 surface markers were assessed via flow cytometry in four Control, four CD133-KO, and five TRIM28-KO Caco2 clones. Based on the obtained data, all analyzed markers were categorized into three groups according to their expression patterns across CD133-KO and TRIM28-KO phenotypes, as well as their overall detectability in the Caco2 clones (Figure 5).

The largest group consisted of markers that showed no detectable expression (relative fluorescence intensity, RFI < 2) in any of the 14 Caco2 clones analyzed. This group included CD31, CD34, CD73, CD90, CD117, CD152, CD200, CD273, CD279, CD282, CD283, CD284, CD325, and calreticulin. Interestingly, eight of these markers—CD34, CD90, CD152, CD200, CD273, CD279, CD284, and CD325—also exhibited no expression at the mRNA level in any of the clones (TPM < 1), whereas the remaining six genes were transcriptionally detectable in at least one clone (Figure 5, Supplementary Table S2): several of them exhibited statistically significant differences in expression in the TRIM28-KO vs. Control and TRIM28-KO vs. CD133-KO comparison groups, respectively: CD31 (~6.0-fold decrease; FDR < 0.001/~7.7-fold decrease; FDR < 0.001), CD73 (~2.9-fold increase; FDR < 0.001/~3.1-fold increase; FDR < 0.001), CD117 (~13.4-fold increase; FDR < 0.001/not significant), CD282 (both not significant), CD283 (~4.2-fold increase; FDR = 0.005/~4.2-fold increase; FDR = 0.005) and calregulin (both not significant).

The second group included markers that were expressed in at least one clone but did not show differential expression across the studied groups. These included CD29 (F(2,10) = 2.01, *p* = 0.18), CD44 (F(2,10) = 1.29, *p* = 0.32), CD49b (F(2,10) = 0.18, *p* = 0.84), CD54 (F(2,10) = 0.86, *p* = 0.45), CD56 (F(2,10) = 0.44, *p* = 0.46), CD112 (F(2,10) = 0.28, *p* = 0.76), CD166 (F(2,10) = 0.48, *p* = 0.63), CD324 (F(2,10) = 0.35, *p* = 0.71), and CD326 (F(2,10) = 0.98, *p* = 0.41). Only CD166 expression at mRNA level was shown statistically significant in both TRIM28-KO vs. Control and TRIM28-KO vs. CD133-KO groups (~9.9-fold increase; FDR < 0.001/~11.0-fold increase; FDR < 0.001).

The smallest group comprised markers whose expression patterns showed potentially interesting trends in association with the TRIM28-KO phenotype, despite lacking statistical significance. Specifically, CD24, CD47, and CD274 demonstrated expression profiles suggestive of correlation with TRIM28 loss in Caco2 clones (Figure 5A,B). Although none of these markers reached statistical significance, the mean CD24 expression was elevated in the TRIM28-KO group. However, high inter-clonal variability within the Control group—due to elevated CD24 expression in clone S5—may have masked potential differences. CD47 expression was markedly increased in TRIM28-KO clones (mean RFI: Control, 6.09; CD133-KO, 5.87; TRIM28-KO, 84.76), though high variance caused by extreme upregulation in clone T50 rendered the difference statistically nonsignificant. Similarly, CD274 was detectable only in two TRIM28-KO clones (T10 and T14). Notably, both CD47 and CD274 were upregulated only in TRIM28-KO clones that also exhibited a decrease in CD133 expression—T10 and T14 for both markers, and T50 for CD47—suggesting that the simultaneous loss of TRIM28 and downregulation of CD133 may be required to modulate the expression of these immune-associated surface proteins. Regarding the mRNA expression of these markers, CD47 was significantly upregulated in TRIM28-KO clones in both the TRIM28-KO vs. Control and TRIM28-KO vs. CD133-KO comparison groups (~3.9-fold increase; FDR = 0.001/~ 5.7-fold increase; FDR < 0.001). In addition, CD24 expression was elevated in TRIM28-KO clones only in the TRIM28-KO vs. CD133-KO group (~5.8-fold increase; FDR < 0.001). CD274 mRNA expression was detected exclusively in clone T10 (TPM = 2.9), which also exhibited the highest protein-level expression. Collectively, these mRNA data corroborate the observed trends of increased expression for all three markers CD24, CD47, and CD274 upon TRIM28 knockout.

Moreover, we verified the impact of TRIM28 knockout on CD133 expression. The analysis confirmed a significant decrease in CD133 levels in TRIM28-KO clones (F(2,10) = 24.34, *p* = 0.0001). Tukey’s post hoc test revealed significant differences between Control and CD133-KO (*p* = 0.0002), as well as between Control and TRIM28-KO (*p* = 0.0002). Regarding CD133 mRNA expression, no statistically significant reduction was observed in the TRIM28-KO group. This was primarily due to one clone, T47, which did not exhibit any decrease at either the protein level (RFI = 12,426) or the mRNA level (TPM = 232). However, the mRNA expression levels in the remaining TRIM28-KO clones T10, T14, and T50 were notably lower (mean TPM = 27.0 ± 19.2) compared to those in the Control group (mean TPM 97.9 ± 32.9) and the CD133-KO group (mean TPM: 100.5 ± 28.1). Notably, among the 27 surface markers analyzed at the protein level, CD133 was the only marker to exhibit a consistent and statistically significant response to TRIM28 loss, highlighting its unique sensitivity to TRIM28-dependent regulatory mechanisms.

TRIM28 protein expression was assessed by Western blot analysis in four CD133-KO and in four Control Caco2 clones. Representative Western blot images are shown in Supplementary Figure S1. Quantitative analysis revealed that TRIM28 expression was reduced by approximately 1.47-fold in CD133-KO clones compared to Control clones (mean values: 2.69 for Control vs. 1.83 for CD133-KO; Student’s *t*-test, t(6) = 2.53, *p* = 0.045). Although the observed reduction was statistically significant, the modest fold change and *p*-value near the significance threshold warrant cautious interpretation. These findings may indicate a potential reciprocal regulatory effect of CD133 knockout on TRIM28 expression.

Hereby, surface marker profiling in CD133- and TRIM28-deficient Caco2 clones revealed that, among 27 markers analyzed, only CD133 consistently responded to TRIM28 loss with a statistically significant decrease in protein expression. Most other markers showed no differential expression or were undetectable. However, CD24, CD47, and CD274 demonstrated expression trends suggestive of correlation with TRIM28 knockout—particularly in clones that also exhibited reduced CD133 levels. Although these differences were not statistically significant at the protein level, RNA-Seq data confirmed strong transcriptional upregulation of CD24 and CD47 in TRIM28-KO clones, which is encouraging and supports their potential involvement in TRIM28-dependent regulatory pathways. Interestingly, TRIM28 protein levels were modestly but significantly reduced in CD133-KO clones, suggesting a possible reciprocal regulatory relationship between TRIM28 and CD133.

## 3. Discussion

In this study, we conducted a comparative molecular and functional study of Caco2 colorectal cancer cell clones with individual knockouts of either CD133, a widely used CSC surface marker [5,9], or TRIM28, an epigenetic regulator previously shown to modulate CD133 expression [15]. Strikingly, CD133 knockout had no impact on the key cellular properties, including proliferation, drug resistance, and global gene expression. In contrast, TRIM28 knockout induced profound changes, including reduced proliferation, enhanced resistance to multiple chemotherapeutic agents, extensive transcriptional reprogramming characterized by upregulation of mitogenic and immune-related genes and alterations in cell surface markers expressions. Notably, loss of TRIM28 consistently downregulated CD133 protein levels, while CD133 mRNA levels remained unaffected, suggesting a post-transcriptional regulatory mechanism.

Interestingly, although we previously observed differences in proliferative activity between Caco2 cells exhibiting CD133-/low and CD133+/high phenotypes [15], the knockout of CD133 did not affect cell proliferation, nor did it alter other functional or phenotypic characteristics of the cells. Our results suggest that CD133 may serve as a phenotypic correlate rather than a functional driver of CSCs traits in Caco2 cells. Moreover, impact of CD133 knockout may depend strongly on the specific cellular model. For example, in Huh7 hepatocellular carcinoma cells, CD133 knockdown impaired proliferation, reduced ATP production, and suppressed tumorigenic capacity, suggesting a pro-survival role [17]. Conversely, in synovial sarcoma, CD133- cells demonstrated increased growth and AKT activity, implying an inhibitory function for CD133 in that context [18]. In addition, in HT29 cells with CD133 knockdown, as well as in CD133+ sorted populations from SW480 cells, no differences in proliferation were observed [19]. Nonetheless, in both models, CD133+ populations exhibited increased resistance to the apoptosis inducer staurosporine and enhanced migratory capacity, suggesting a context-dependent role of CD133 in promoting survival and motility rather than proliferation. Collectively, these observations suggest that the phenotypic and functional outcomes of CD133 knockout are highly dependent on the cellular context, underscoring the tissue-specific and model-dependent nature of CD133-associated regulatory mechanisms in cancer. It is also important to note that CD133 knockout may activate compensatory mechanisms within the cell that mitigate the loss of CD133 function, potentially obscuring its direct biological impact. Nevertheless, knockout of TRIM28, a known regulator of CD133 expression, resulted in pronounced phenotypic alterations, indicating that upstream modulators of CD133 may exert broader and more direct effects on cellular behavior.

First, we demonstrated that TRIM28 knockout in Caco2 colorectal cancer cells results in a significant reduction in proliferation rate, as evidenced by delayed monolayer formation during live-cell imaging (Figure 1). This finding aligns with extensive data implicating TRIM28 as a direct regulator of tumor cell proliferation across multiple cancer types, with numerous studies reporting a reduction in proliferative activity following its depletion [20,21,22,23]. Interesting, that our data also suggest that proliferation-based metrics such as mitotic index (MI) may not fully capture functional changes, especially in the context of TRIM28 loss. Despite significantly delayed proliferation, MI remained unchanged between all three groups: Control, CD133-KO and TRIM28-KO. This emphasizes the need for multiparametric assessment, including direct live-cell imaging (Figure 1) or cell division-associated markers expression (Figure 4), when evaluating proliferative dynamics in cancer models. Importantly, all of the top ten enriched Gene Ontology (GO) Biology Processes terms were directly associated with cell division, and part of Molecular Function and Cellular Component terms further highlighting the involvement of TRIM28 in the regulation of proliferative programs (Figure 4D–F). The divergence between confluence-based proliferation assessment and mitotic index (MI) suggests that these parameters capture distinct aspects of cell growth. This discrepancy may be explained by the fact that MI does not capture the full dynamics of the cell cycle or proliferation rate, as mitosis is a brief phase relative to the entire cycle. Thus, MI alone is insufficient for precise proliferation assessment and should be combined with other markers for robust analysis [24,25,26]. However, confluence-based measurements depend not only on the rate of cell division but also on cell morphology, adhesion, and survival. For instance, increased cell size or spreading can delay apparent confluence without affecting mitotic activity, whereas reduced attachment or increased cell death may similarly alter surface coverage [27,28]. Thus, the slower proliferation inferred from confluence imaging in TRIM28-KO clones may arise from changes in cytoskeletal organization, adhesion strength, or contact inhibition rather than from direct alterations in cell-cycle progression.

Previous studies have demonstrated that TRIM28 modulates the expression of actin-binding and adhesion-related genes, supporting the possibility that its loss affects cell spreading and substrate interactions [29,30]. Consistent with these reports, our transcriptomic analysis revealed the upregulation of several genes involved in cytoskeletal remodeling and adhesion, including ARHGAP44, ZDHHC2, CDH11, and ITGAV (Figure 4E,F). Therefore, the observed inconsistency between confluence-based proliferation and MI likely reflects multifactorial changes encompassing cell morphology, size, and adhesion, rather than a simple reduction in mitotic activity. A combined assessment of cell area, viability, and cytoskeletal architecture would help to clarify these contributions in future studies.

Interestingly, not only does CD133 knockout fail to affect cell migratory activity, but TRIM28 knockout also shows no impact (Figure 1B,F), despite previous studies reporting that reduced TRIM28 expression correlates with decreased migratory potential [29,31,32]. Interestingly, several GO terms associated with cell migration were enriched (Figure 4); however, the phenotypic alterations induced by TRIM28 knockout were apparently insufficient to produce detectable changes in migration in the scratch assay. Moreover, our findings could be explained by the cell line–specific biology of TRIM28, whose function in Caco2 cells does not appear to be critical for the regulation of migration-related properties. It is possible that TRIM28 plays no role in EMT or migration in certain cellular contexts such as Caco2, or that its function is highly dependent on specific signaling pathways. These observations are further supported by the fact that the expression levels of E- and N-cadherins, main markers of EMT, remain unchanged following TRIM28 knockout: E-cadherin expression persists at consistent levels, while N-cadherin remains undetectable both on mRNA and protein levels (Figure 5, Supplementary Table S3).

Interestingly, our findings (Figure 2) showing increased resistance of TRIM28-KO clones to several chemotherapeutic agents appear to contradict the majority of studies reporting that TRIM28 suppression sensitizes tumor cells to treatment [23,33,34]. However, in gastric cancer, TRIM28 suppressed CSC like characteristics via Wnt/β-catenin signaling and its knockdown enhanced the proliferation and clonogenic capacity of gastric cancer cells. Moreover, TRIM28 silencing upregulated stemness-associated markers and promoted both spheroid formation and drug resistance in these cells [35]. Apparently, our findings can be explained by the lower sensitivity of cells with reduced proliferative activity to cytostatic agents, which is supported by previously published data [36,37].

We did not observe any effect of CD133 or TRIM28 knockout on the mitochondrial membrane potential of Caco2 cells, the percentage of cells undergoing spontaneous apoptosis, or their sensitivity to staurosporine (Figure 3). It is worth noting that existing studies report contradictory findings regarding the correlation between CD133 expression and mitochondrial potential [38,39]; however, the majority of available data support a positive correlation between CD133 expression and resistance to apoptosis [40]. Although substantially fewer studies have examined the relationship between TRIM28 and mitochondrial membrane potential, the available data consistently support a positive correlation between this transcriptional factor and mitochondrial potential [41,42]. Furthermore, a larger body of evidence indicates that TRIM28 expression positively correlates with apoptosis resistance to apoptosis across various cell types, including cancer cells [43].

Our transcriptomic analysis revealed that TRIM28 knockout, but not CD133 depletion, induces substantial changes in gene expression in Caco2 colorectal cancer cells (Figure 5). Principal component analysis clearly segregated TRIM28-KO clones from both Control and CD133-KO groups, which formed a single transcriptional cluster. Consistently, differential expression analysis identified hundreds of TRIM28-regulated genes, many of which were shared between both TRIM28-KO vs. Control and TRIM28-KO vs. CD133-KO comparisons, confirming the robustness of the observed transcriptional shifts.

To further illustrate these changes, we examined specific examples of TRIM28-dependent transcripts. Notably, loss of TRIM28 led to derepression of endogenous retroviruses, generating dsRNA that stimulates interferon signaling [44]. Among these, ERVV-1 (~77.2-fold increase; FDR < 0.001) and ERVV-2 (~141.9-fold increase; FDR < 0.001) were found to be upregulated in TRIM28-KO cells, supporting the role of TRIM28 in ERV silencing and antiviral regulation. In addition, the interferon-stimulated gene IFI44L [45] was markedly upregulated (~478.0-fold increase; FDR < 0.001) in TRIM28-KO cells, further indicating activation of antiviral pathways. Together, these findings highlight a link between TRIM28 loss, ERV derepression, and enhanced interferon signaling.

Moreover, consistent with previous findings, ZNF354C (~29.1-fold increase; FDR < 0.001) has been shown to interact directly with TRIM28 through its KRAB domain, forming a repressive complex that mediates transcriptional silencing of endothelial target genes [46]. It should be noted that TRIM28 acts as a universal co-repressor for KRAB-C2H2 zinc finger proteins (KRAB-ZNFs) by binding their conserved KRAB domains and recruiting chromatin-modifying enzymes such as SETDB1, HP1, and HDACs [47,48]. This conserved mechanism underlies broad functional associations between TRIM28 and most KRAB-ZNFs. In our dataset, more than 50 genes encode KRAB-ZNF proteins, including ZNF699, ZNF613, ZNF559, ZNF675, and others, which are therefore likely TRIM28 partners. Collectively, these factors form an extensive regulatory network that links DNA sequence recognition by ZNFs with TRIM28-mediated chromatin silencing and transcriptional repression.

Gene Ontology enrichment highlighted a strong association of TRIM28 loss with cell cycle regulation, with the top enriched categories involving mitotic processes, centromere architecture, and midbody formation. Interestingly, upregulated genes included both positive and negative regulators of proliferation, suggesting a complex compensatory response to TRIM28 depletion. These findings align with prior studies implicating TRIM28 in the maintenance of proliferative programs in cancer cells and provide transcriptomic-level support for its role as a key regulatory hub [20,21,22,23].

Importantly, TRIM28 knockout also induced marked changes in genes associated with membrane localization, adhesion, and migration, particularly when compared to CD133-KO clones (Figure 5). Enriched GO terms included categories such as cadherin binding, actomyosin assembly, and EMT consistent with the observed upregulation of EMT drivers (e.g., SNAI2, TGFB2, TGFBR1) and matrix remodeling factors (e.g., SPARC, THBS1, WNT7A). Notably, CD133 knockout had no detectable impact on transcriptomic profiles, suggesting its dispensability for baseline gene expression in Caco2 cells. Therefore, the comprehensive DEG signatures identified in TRIM28-KO clones likely represent direct or indirect consequences of TRIM28 loss relative to the wild-type transcriptome. Together, these results underscore the central role of TRIM28 in regulating proliferation and migratory phenotypes in colorectal cancer cells and establish a transcriptional foundation for further investigation of its downstream effectors.

In addition, our findings demonstrate that knockout of TRIM28 in Caco2 colorectal cancer cells is associated with a pronounced upregulation of immune checkpoint molecules CD47 and CD24 on both mRNA and protein levels, particularly in clones exhibiting concurrent downregulation of CD133. These results suggest a potential role for TRIM28 in modulating immune evasion mechanisms via regulation of surface “don’t eat me” signals that inhibit macrophage-mediated phagocytosis.

Accumulating evidence supports a functional link between TRIM28 and immune checkpoint regulators such as CD47 and CD24. TRIM28 acts as a key transcriptional co-repressor involved in epigenetic regulation and has been implicated in shaping the tumor immune microenvironment by controlling gene expression programs relevant to immune escape [49]. In murine models of pancreatic necrosis, TRIM28 was shown to regulate CD47 expression through modulation of miR-133a, whereby TRIM28 depletion led to increased miR-133a levels, suppressed CD47 expression, and enhanced macrophage-mediated clearance of tumor cells [50]. These findings are consistent with our observations, suggesting that TRIM28 may directly contribute to the transcriptional control of CD47 and its immune regulatory functions.

In addition to CD47, TRIM28 knockout also led to elevated expression of CD24, a glycoprotein that interacts with Siglec-10 on macrophages and often cooperates with CD47 to promote immune evasion [51]. This upregulation may represent a compensatory response to maintain immune suppression in the absence of TRIM28, particularly in aggressive tumor subtypes exhibiting CSCs phenotypes. Notably, previous reports have demonstrated that TRIM28 supports both stemness and proliferation in such contexts [52], further supporting its role in coordinating immune escape and tumor progression.

Moreover, TRIM28 is known to intersect with multiple signaling cascades—including PI3K/AKT/mTOR and EMT-associated regulators—which are also implicated in the modulation of immune checkpoint expression and CSCs maintenance [53]. In our study, transcriptomic profiling revealed enrichment of gene sets involved in membrane trafficking and cytoskeletal remodeling (Figure 5), suggesting that the concomitant increase in CD47 and CD24 expression may reflect broader reprogramming of cellular adhesion and surface protein localization.

Within the tumor microenvironment, the combined upregulation of CD47 and CD24 likely amplifies “don’t eat me” signaling, thereby enhancing resistance to macrophage-mediated phagocytosis and facilitating immune evasion [54,55]. Collectively, our findings indicate that targeting TRIM28 may exert complex effects on tumor–immune interactions through modulation of key checkpoint molecules, underscoring the need for further mechanistic studies to elucidate the role of TRIM28 in immune escape within colorectal cancer.

Pathway-level analysis confirmed that TRIM28 loss induces robust and reproducible signaling alterations in Caco2 cells. In both TRIM28-KO versus Control and TRIM28-KO versus CD133-KO comparisons, we observed consistent activation of JAK/STAT, TNFα, and NFκB pathways, alongside moderate upregulation of p53, WNT, TGFβ, and hypoxia signaling. These findings are in line with previous studies implicating TRIM28 in the regulation of cytokine and inflammatory signaling, particularly through JAK/STAT- and NFκB-dependent transcriptional programs [56,57,58]. Concomitantly, proliferative cascades such as EGFR, PI3K, and MAPK were uniformly downregulated, which is consistent with reports describing TRIM28 as a positive regulator of cell cycle progression and growth factor–mediated proliferation in various cancers [21,22].

The observed pathway shifts are strongly concordant with our transcriptomic results (Figure 4), where TRIM28 knockout enriched for GO terms related to cell division, membrane localization, and adhesion. Functionally, these alterations mirror the reduced proliferation (Figure 1) and increased chemoresistance (Figure 2) of TRIM28-KO clones, both of which may be explained by suppression of EGFR/PI3K/MAPK activity. Indeed, growth factor signaling via EGFR and downstream MAPK/PI3K cascades is a critical driver of cell proliferation and drug sensitivity in colorectal cancer [59,60], and its attenuation is consistent with the reduced responsiveness of TRIM28-KO cells to cytotoxic agents.

In parallel, activation of JAK/STAT and NFκB pathways may contribute not only to the altered proliferative phenotype but also to immune escape. NFκB has long been recognized as a central regulator of inflammatory signaling and immune checkpoint expression [61,62], while JAK/STAT cascades intersect with both cytokine signaling and cancer progression [63,64]. In our study, TRIM28-KO clones demonstrated transcriptional upregulation of CD24 and CD47 (Figure 5), two well-established “don’t eat me” signals that suppress macrophage-mediated phagocytosis [42,45]. Previous reports also support a functional interplay between TRIM28 and immune checkpoint molecules, including regulation of CD47 via epigenetic and miRNA-dependent mechanisms [41]. Thus, the simultaneous activation of NFκB and JAK/STAT signaling in TRIM28-deficient cells provides a plausible mechanistic link to enhanced expression of CD24/CD47, suggesting that TRIM28 modulates tumor–immune interactions through coordinated regulation of cytokine and adhesion pathways.

Importantly, the concordance of both up- and downregulated pathways across the TRIM28-KO versus Control and TRIM28-KO versus CD133-KO comparisons indicates that these changes are specific to TRIM28 deficiency rather than clonal variation. Although subtle differences were noted—such as stronger activation of JAK/STAT and TNFα in the Control comparison and more pronounced suppression of MAPK and estrogen signaling relative to CD133-KO—the overall overlap was substantial. Together, these results emphasize TRIM28 as a central regulator that shifts the balance from growth factor–driven proliferation (EGFR/PI3K/MAPK) toward cytokine- and stress-related signaling (JAK/STAT, NFκB, TNFα), thereby integrating proliferative control with immune modulation in colorectal cancer cells.

In summary, our study highlights TRIM28 as a multifaceted regulator of colorectal cancer cell behavior, integrating control over proliferation, chemoresistance, and immune evasion. While CD133 knockout had no impact on cellular or molecular phenotypes, TRIM28 depletion induced pronounced transcriptomic and functional alterations, including the upregulation of CD47 and CD24, key immune checkpoint molecules. These findings underscore TRIM28 as a potential therapeutic target and warrant further investigation into its context-dependent roles in cancer progression and immune modulation.

## 4. Materials and Methods

### 4.1. Cell Culture

Caco2 clones with TRIM28 knockout (TRIM28-KO) and reference control clones (Control) [15] were provided by the Laboratory of Cell Biology, Institute of Biomedical Chemistry (Moscow, Russia). CD133 knockout (CD133-KO) Caco2 clones were generated using a previously described protocol [16]. All cell lines were cultured in DMEM/F-12 medium (1:1) supplemented with 10% fetal bovine serum (FBS; Thermo Fisher Scientific, Waltham, MA, USA) and 2 mM L-glutamine. Cells were maintained at 37 °C in a humidified 5% CO_2_ atmosphere and used for experiments within ten passages after thawing. Routine mycoplasma testing was performed using the MycoReport kit (Evrogen, Moscow, Russia). Caco2 cell line identity was confirmed by short tandem repeat (STR) profiling.

### 4.2. Cell Proliferation Assay

Cells were seeded in 12-well plates at a density of 5 × 10^4^ cells per well. Proliferation dynamics were monitored using the Incucyte ZOOM live-cell imaging system (Sartorius, Göttingen, Germany), with image acquisition every 2 h using a 20× objective. Image analysis was performed using Incucyte Software (version 2016A), applying the following segmentation parameters: adjustment factor = 1.3, hole fill threshold = 3000 µm^2^, and minimum object size = 500 µm^2^. Cells were considered confluent at 95% coverage.

### 4.3. Cell Migration Assay

Cells were seeded into ImageLock 96-well plates (Sartorius) at a density of 2 × 10^5^ cells per well. Following 24 h incubation, cell proliferation was inhibited with 10 μg/mL mitomycin C (Merck, Rahway, NJ, USA) for 2 h. Wounds were introduced using the Incucyte 96-pin WoundMaker tool (Sartorius, Göttingen, Germany). Cell migration was tracked every 1.5 h under a 20× objective. Image analysis was conducted using the same software and parameters as in the proliferation assay.

### 4.4. Flow Cytometry (FACS)

Cells were detached using trypsin and washed twice in PBS (PanEco, Moscow, Russia) containing 1% FBS (Gibco, Waltham, MA, USA). Surface antigens were labeled with fluorophore-conjugated antibodies (Table 1) at manufacturer-recommended concentrations for 20 min at 4 °C in the dark. Cells were subsequently fixed using BD Cytofix (BD Biosciences, Franklin Lakes, NJ, USA), except for phosphatidylserine staining, which was performed on live cells with the addition of SYTOX Blue (Thermo Fisher Scientific). Acquisition was performed on a BD FACSAria III cytometer, with a minimum of 10^4^ events collected per sample. Data were analyzed in FlowJo_V10 (FlowJo™, Ashland, OR, USA), and relative fluorescence intensity (RFI) was calculated as the ratio of median intensity of specific fluorescence from antibody-labeled cells to the median intensity of autofluorescence of unstained control cells.

### 4.5. Imaging Flow Cytometry and Mitotic Index Calculation

Imaging flow cytometry was conducted using the Amnis ImageStreamX Mk II system (Luminex Corp., Austin, TX, USA). Data analysis was performed with IDEAS 6.2 software (Cytek Biosciences, Fremont, CA, USA). Cells were fixed in BD Cytofix buffer and nuclear staining was performed by Hoechst 33342 (Thermo Fisher Scientific) with final concentration of 10 µg/mL. Focused cells were gated based on the Gradient RMS parameter (brightfield, top 50% of events), followed by gating singlets based on “Area” (<1000 µm^2^) and “Aspect Ratio” (>0.7). Mitotic cells were defined as focused singlet cells with Bright Detail Intensity R3 values exceeding 2.35 standard deviations above the population mean along with algorithm described previously for Caco2 cell line [65]. MI was calculated as the proportion of mitotic cells the total number of focused singlet cells.

### 4.6. Western Blotting

Cells were lysed in 90 μL of RIPA buffer (150 mM NaCl, 1% Triton X-100, 0.5% sodium deoxycholate, 0.1% SDS, 50 mM Tris-HCl, pH 8.0) for 15 min on ice. Lysates were supplemented with Laemmli buffer (1× final) and 5% 2-mercaptoethanol, heated at 95 °C for 10 min, and stored at −80 °C. Proteins (50 µg per sample) were separated on 14% SDS-PAGE and transferred to nitrocellulose membranes (Bio-Rad, Hercules, CA, USA). Membranes were blocked in EveryBlot blocking buffer (Bio-Rad) followed by incubation with mouse monoclonal anti-TRIM28 (1:1000, clone 20C1, ab22553; Abcam, Cambridge, England, UK), mouse monoclonal anti-alpha-tubulin antibodies (1:1000, clone DM1A, T6199; Sigma-Aldrich, St. Louis, MO, USA) and HRP-conjugated secondary antibodies for 1 h. Protein bands were visualized using ECL substrate (Bio-Rad), and chemiluminescent signals were detected with a DNR LumiBis Gel Imaging System 3.2 using GelCapture software (DNR Bio-Imaging Systems Ltd., Aachen, Germany). ImageJ (software version 1.54p; February 2025) was employed for densitometry and quantification.

### 4.7. Mitochondrial Membrane Potential Assessment

Cells were seeded at 2 × 10^5^ per 35 mm dish and stained after 48 h with 100 nM MitoView633 (Biotium, Fremont, CA, USA) for 15 min at 37 °C. Cells were then detached, washed in PBS, and analyzed by flow cytometry using the Alexa Fluor 700 channel (633 nm laser) on BD FACSAria III. Relative fluorescence intensity (RFI) values were used for quantification of mitochondrial activity (see Section 4.4).

### 4.8. MTT Cytotoxicity Assay

A colorimetric MTT assay was used to assess the viability of different Caco2 clones following treatment with five cytotoxic compounds: staurosporine, doxorubicin, paclitaxel, cisplatin, and curcumin (all from Sigma-Aldrich). Cells were seeded in 96-well plates at a density of 2 × 10^4^ cells/well and incubated with test compounds in complete culture medium for 48 h. Each concentration was tested in four technical replicates. Following treatment, the medium was gently removed and cells were incubated with 30 µL of 0.5 mg/mL MTT reagent (PanEco) for 2 h at 37 °C to allow formation of formazan crystals. Subsequently, 100 µL of DMSO (PanEco) was added to dissolve the crystals. Absorbance was measured at 565 nm using a Tecan Infinite M200 Pro plate reader (Tecan, Männedorf, Switzerland). Viability was expressed as a percentage relative to untreated control cells.

### 4.9. RNA Isolation, Library Preparation, and Sequencing

Total RNA was extracted from 3 × 10^6^ cells derived from Caco2 TRIM28-KO clones, CD133-KO clones, and reference clones using the RecoverAll Total Nucleic Acid Isolation Kit (Thermo Fisher Scientific), according to the manufacturer’s instructions. RNA concentration and integrity were assessed using the Agilent RNA 6000 Nano Kit (Agilent Technologies, Santa Clara, CA, USA) and the Qubit RNA Assay Kit (Thermo Fisher Scientific). Enrichment of mRNA and construction of sequencing libraries were performed using the KAPA RNA HyperPrep RiboErase Kit (KAPA Biosystems, Wilmington, MA, USA). Library concentration and quality were evaluated using the Qubit RNA HS Assay Kit (Thermo Fisher Scientific) and the Agilent TapeStation system (Agilent Technologies). Sequencing was performed on an Illumina NextSeq 550 system (Illumina, San Diego, CA, USA). A minimum of 30 million raw reads per sample was obtained. Data available on request from the authors

### 4.10. Bioinformatic Analysis

Raw sequencing data were obtained in FASTQ format. Quality control of raw reads was conducted using FastQC (http://www.bioinformatics.babraham.ac.uk/projects/fastqc/ (accessed on 15 June 2025)). Quantification of gene and transcript expression was performed using Salmon (version 1.10.0; 24 February 2023) in mapping-based mode [66]. Transcript abundance was expressed in transcripts per million (TPM), and gene-level expression was calculated by summing TPM values of all transcripts belonging to the same gene. The human reference transcriptome GRCh38 (Ensembl release 113; October 2024) was used. Differential gene expression analysis was conducted using the DESeq2 package [67]. Gene set enrichment analysis was performed using the ClusterProfiler package [68]. A source of biological knowledge, we utilized the Gene Ontology database (biological processes, molecular function and cellular component categories) and PROGENy databases [69]. Genome annotation in GTF format (GRCh38, release 113) was downloaded from Ensembl. The analysis was restricted to protein-coding genes located on standard chromosomes (1–22, X, and Y). Differentially expressed genes (DEGs) were identified using the following thresholds: log2 fold change (log2FC) > 1 for upregulated genes, log2FC < –1 for downregulated genes, and a false discovery rate (FDR) < 0.05.

### 4.11. Statistical Analysis

All statistical analyses and data visualization (with exception of whole transcriptomic analysis) were performed using GraphPad Prism (version 10.4.0; GraphPad Software, San Diego, CA, USA). Specific statistical tests are described in the respective figure legends and Section 2 Values are presented as mean ± standard deviation (SD). A *p*-value ≤ 0.05 was considered statistically significant.

## Figures and Tables

**Table 1:** List of antibodies for FACS analysis used in work.

№	Name	Company	Cat. №
1	anti-CD24-APC	Miltenyi Biotec (Bergisch Gladbach, Germany)	130-095-954
2	anti-CD29-APC	Miltenyi Biotec	130-101-280
3	anti-CD31-BV510	Bd Biosciences (Franklin Lakes, NJ, USA)	563454
4	anti-CD34-FITC	Bd Biosciences	555821
5	anti-CD44-FITC	Miltenyi Biotec	130-113-334
6	anti-CD47-APC	BioLegend (San Diego, CA, USA)	323124
7	anti-CD49b-FITC	Miltenyi Biotec	130-123-686
8	anti-CD54-APC	Miltenyi Biotec	130-121-342
9	anti-CD56-APC	Miltenyi Biotec	130-113-305
10	anti-CD73-PE	Bd Biosciences	550257
11	anti-CD90-FITC	Bd Biosciences	555595
12	anti-CD112-PE	Miltenyi Biotec	130-122-770
13	anti-CD117-APC	Miltenyi Biotec	130-11-541
14	anti-CD133-APC	Miltenyi Biotec	130-113-106
15	anti-CD152-APC	Bd Biosciences	555855
16	anti-CD166-APC	Miltenyi Biotec	130-119-769
17	anti-CD200-APC	BioLegend	399808
18	anti-CD273-APC	BioLegend	345508
19	anti-CD274-PE	Miltenyi Biotec	329706
20	anti-CD279-FITC	BioLegend	329904
21	anti-CD282-FITC	BioLegend	309705
22	anti-CD283-PE	BioLegend	315009
23	anti-CD284-APC	BioLegend	312815
24	anti-CD324-APC	Miltenyi Biotec	130-095-412
25	anti-CD325-APC	BioLegend	350808
26	anti-CD326-APC	Miltenyi Biotec	130-111-000
27	anti-PS-FITC (FITC Annexin V)	BioLegend	640906
28	anti-Calregulin-PE	Santa Cruz Biotechnology (Dallas, TX, USA)	sc-373863

## Data Availability

The data presented in this study are available within the article text, figures, tables, and Supplementary Materials. Raw transcriptome data files are publicly released on the NCBI SRA (https://www.ncbi.nlm.nih.gov/sra/PRJNA1348323, accessed on 24 October 2025). The accession number is PRJNA1348323 for both Control and KO clones.

## Supplementary Materials

The supporting information can be downloaded at https://www.mdpi.com/article/10.3390/ijms262210862/s1.
