# Supplementary figures and images for: Contrasting Impacts of Targeted Disruption of the Cancer Stem Cell Marker CD133 and Its Epigenetic Regulator TRIM28 in Colorectal Cancer Cells
Source: Int J Mol Sci. 2025 Nov 9;26(22):10862. doi: 10.3390/ijms262210862 (PMC12652789; doi:10.3390/ijms262210862)

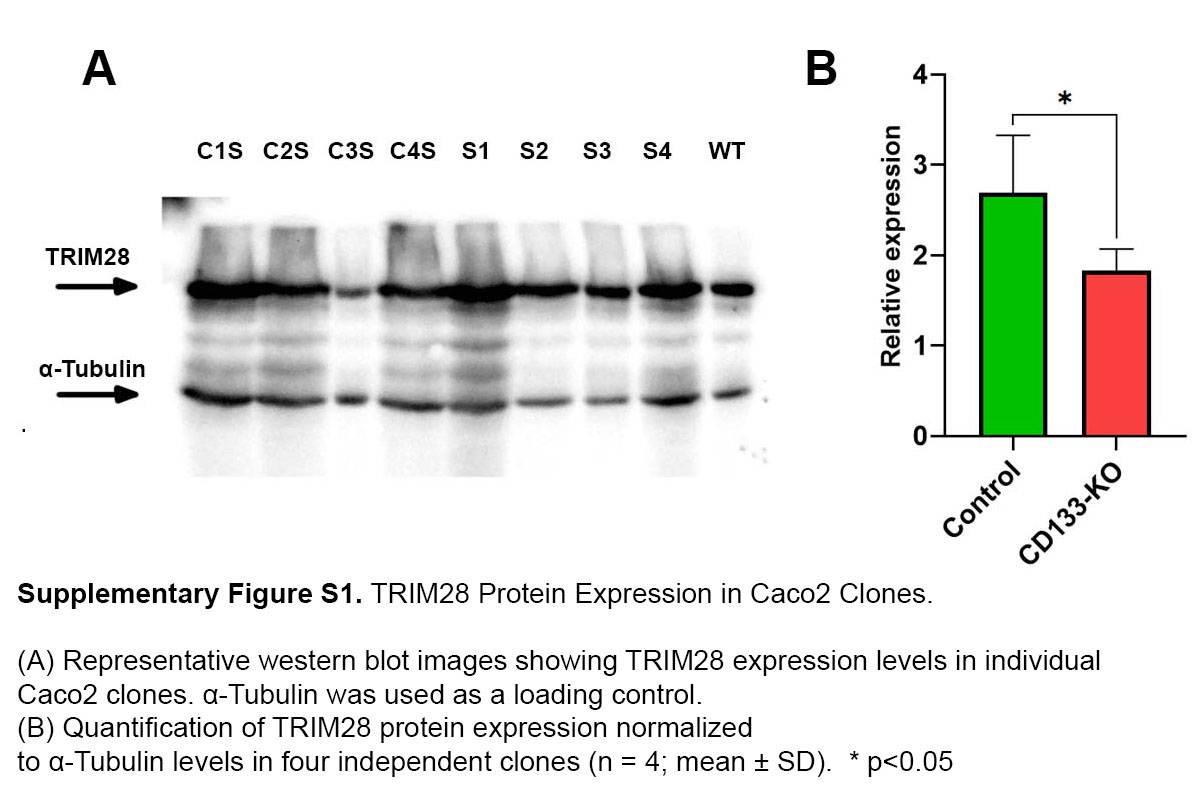

Supplement: Supplementary file 1 [file ijms-26-10862-s001.zip › Supplementary Figure S1 copy.jpg]
